# Essential interventions for maternal, newborn and child health: background and methodology

**DOI:** 10.1186/1742-4755-11-S1-S1

**Published:** 2014-08-21

**Authors:** Zohra S Lassi, Rehana A Salam, Jai K Das, Zulfiqar A Bhutta

**Affiliations:** 1 Division of Women and Child Health, Aga Khan University, Karachi, Pakistan

**Keywords:** Maternal health, newborn health, child health, essential interventions, mortality

## Abstract

Worldwide, 250,000–280,000 women die during pregnancy and childbirth every year and an estimated 6.55 million children die under the age of five. The majority of maternal deaths occur during or immediately after childbirth, while 43% of child death occurs during the first 28 days of life. However, the progress in limiting these has been slow and sporadic. In this supplement of five papers, we aim to systematically assess and summarize essential interventions for reproductive, maternal, newborn and child health from relevant systematic reviews. This paper is an introductory paper detailing the background and methodology used for grading interventions. The following three papers summarize the evidence on essential interventions for pre-pregnancy, pregnancy, childbirth, postnatal (mother and neonatal) and child heath while the last paper describes the essential interventions as per the level of health care delivery and their proposed packages of care.

## Why maternal, newborn and child health?

Poor maternal, newborn and child health care remains a significant problem in low and middle income countries (LMICs). Worldwide, 250,000–280,000 women die during pregnancy and childbirth every year [1] and an estimated 6.55 million children die under the age of five [2]. The majority of maternal deaths occur during or immediately after childbirth. A child’s risk of dying is highest during the first 28 days of life when about 3.5% of under-five deaths take place, translating into 2.85 million deaths [2]. Up to one half of all newborn deaths occur within the first 24 hours of life and 75% occur in the first week. Children in LMICs are nearly 56 times more likely to die before the age of five than children in high-income countries (HICs) [2].

Good maternal health care and nutrition are important contributors to child survival; maternal infections and other poor conditions often contribute to indices of neonatal morbidity and mortality (including stillbirths, neonatal deaths and other adverse clinical outcomes) [3]. Considering the fact that most maternal and child deaths are preventable using current knowledge, the burden of mortality and morbidities is unacceptably high. The majority of maternal deaths occur during labour, delivery, and the immediate postpartum period, with obstetric haemorrhage being the main medical cause of death. Hypertensive diseases, infections, obstructed labour, and abortion-related complications are the other causes of maternal mortality. The maternal mortality ratio is approximately 500 per 100,000 live births in sub-Saharan Africa, compared to around 150 per 100,000 live births in South Asia and 16 per 100,000 live births in HICs [1]. Furthermore, the main direct causes of neonatal mortality and morbidity are infections, complications arising from preterm birth, and intrapartum-related neonatal deaths, which account for nearly 80% of all neonatal deaths globally [4]. Almost (99%) all maternal, newborn, and child deaths occurs in LMICs, unquestionably, appropriate interventions along with appropriate health resources in these countries have significant potential for reducing the burden of maternal and child mortalities [5,6]. Although substantial progress has been made towards achieving the Millennium Development Goals (MDGs) 4 and 5, the rates of decline in maternal, newborn and under-five mortality remain insufficient to achieve these goals by 2015 [3]. Furthermore, progress is marked by larger inequities, not only across regions and countries, but also within countries where maternal and child mortality rates and health care indicators differ substantially by geographic location (higher in rural areas versus urban areas) as well as by socioeconomic status.

While many factors contribute to maternal and neonatal deaths, one of the effective means of reducing this burden is provision of effective preventive measures or early treatment provided to women and newborns, often at their home or in primary health care settings. Worldwide 50 million births take place at home without a skilled birth attendant (SBA) [7]. The rates of no access to skilled birth care and emergency obstetric care are higher in LMICs where majority of deaths and morbidity related to complications of childbirth take place [8]. Skilled attendance at birth remains particularly low in sub-Saharan Africa and southern Asia and there are wide disparities within countries, across socio-economic status, geographic location, and educational status. In sub-Saharan Africa, women are alone, with no attendant in more than half of home births while in South Asia, around one-third of home births are without traditional birth attendants. Therefore, effective interventions and improved coverage in low-resource settings have an enormous potential to avert maternal and neonatal deaths. Haemorrhage, contributing to 35% of maternal deaths, rapidly leads to death without intervention, but with simple interventions like blood transfusions, oxytocics to prevent bleeding, and/or manual removal of the placenta by a SBA, severe bleeding can be averted in time to prevent mortality [6,9]. Similarly, access to antenatal health visits and medicines can prevent death from hypertensive disorders, while death due to sepsis can be averted by screening for prenatal maternal infection and sexually transmitted infections (STIs) during antenatal visits and with hygienic infection control measures during birth provided by SBA. Other direct causes of maternal deaths, including obstructed labour, complications of anaesthesia or caesarean section, and ectopic pregnancy, can be prevented with access to antenatal care, skilled birth attendance, and basic and comprehensive emergency obstetric care.

Interventions to avert maternal mortalities can also prevent neonatal deaths; evidence suggests that 77% of all neonatal deaths occurs where the coverage of skilled birth attendance is 50% or even less [10]. Hygienic births through skilled birth attendance can largely prevent neonatal infections through simple treatments such as cleansing of the umbilical cord, and promotion of early and exclusive breastfeeding. Furthermore, providing birth attendants with simple equipment and training is a low-tech, low-cost opportunity to prevent neonatal deaths. Complications from preterm birth and low birth weight (LBW) take the largest toll on neonatal deaths, with more advanced care being required for those born before 33 weeks’ gestation. Use of low cost interventions such as kangaroo mother care (KMC) yields a 51% reduction in mortality for newborns weighing less than 2000g [10,11]. Among children under the age of five years, infection is the major cause of severe morbidity and mortality. Simple interventions such as proper nutrition, sanitation, hygiene, complete and timely immunization along with preventive and therapeutic interventions for the management of diarrhoea and pneumonia can save a major portion of these preventable under-five deaths. According to the recent estimates, scaling up of these key evidence-based interventions coverage to at least 80% and that for immunization to at least 90%, can eliminate 95% of diarrhoea and 67% of pneumonia deaths in children younger than 5 years by 2025 at a cost of $6·715 billion [12].

The current burden of maternal, neonatal and child mortalities, heavily concentrated in LMICs, is especially grave in the light of existing simple, cost-effective and low-technology interventions. Interventions and strategies for improving reproductive, maternal, newborn and child health care (RMNCH) and survival are closely related and must be provided through a continuum of care approach. When linked together and included as integrated programs, these interventions can lower costs, promote greater efficiencies, and reduce duplication of resources. However, few efforts have been made to identify synergies and integrate these interventions across the continuum of care. Despite of the existing plethora of knowledge, there is a lack of consensus on how best to move forward in a coordinated manner so as to achieve progress towards the MDG’s. Furthermore consensus is also needed on the level of evidence [3]. The foremost aim of this global review is to compile existing evidence on the impact of various maternal, newborn and child interventions on the major causes of maternal, newborn and under five deaths. The specific objectives of this review were to serve as a first step towards: developing consensus on the content of RMNCH packages of interventions at each level of the health system across the continuum of care; facilitating the scaling-up of these interventions; and identifying research gaps in the content of core packages of interventions.

## Methodology

### Search strategy

A total of 142 RMNCH interventions were identified, assessed and selected for this review, based on current World Health Organization (WHO) recommendations contained in the following publications: Guidelines on HIV and Infant Feeding (2010) [13]; Integrated Management of Childhood Illness (2008) [14]; Integrated Management of Childhood Illness for High HIV Settings (2008) [15], the Pocketbook on Hospital Care For Children (2005) [16], Recommended interventions for improving maternal and newborn health - Integrated management of pregnancy and childbirth (2007) [17]. Interventions published in the Child and Neonatal Lancet Series (2003 and 2005, respectively) [18,19] (**Refer Figure 1 for essential interventions**). We further updated the evidence on these interventions from Lancet Diarrhoea and Pneumonia Series (2013) [20] and Lancet Maternal and Newborn Nutrition Series (2013) [21].

### Selection and inclusion of Interventions

The interventions were prioritized according to the following criteria:

• Interventions expected to have a **significant impact on maternal, newborn and child survival**, addressing the main causes of maternal, newborn and child mortality.

• Interventions suitable for implementation in **low- and middle-income countries**; minimal essential care.

• Interventions delivered through the **health sector**, from the community up to the 1st referral level of health service provision.

Relevant reviews for each intervention were identified from the following electronic databases: the Cochrane database of systematic reviews, the Cochrane database of abstract reviews of effectiveness (DARE), the Cochrane database of systematic reviews of randomized control trials (RCT’s), and PubMed. The reference lists of the reviews and recommendations from experts in the field were also used as sources to obtain additional publications. The principal focus was on the existing systematic reviews and meta-analysis.

### Classification of interventions

The interventions were classified into categories A, B and C, according to the framework provided in Table 1.

**Table 1:** Classification of interventions according to evidence and delivery strategies

Category	Evidence for intervention GRADE categories	Delivery strategies	Action
**A**	Intervention evidence agreed	Delivery strategy agreed	Disseminate for rapid scale up

**B**	Intervention evidence agreed	Delivery strategy no consensus	Collate evidence and define gaps in evidence for delivery strategies – seek consensus

**C**	Intervention evidence still questioned	Delivery strategy no consensus	Prioritise action and further research required or delete

The classification of the effect of interventions according to the evidence available was done based on that used by the Cochrane group. This classification benefited from being broadly known, recognized and accepted since it is the classification used by the Cochrane systematic review process that has guided this exercise from the beginning. The "evidence" was restricted to published systematic reviews; not including single studies, but to list single studies as background information for further review. Table 2.

**Table 2:** 

A	B	C	D	E
Interventions that are beneficial	Interventions likely to be beneficial	Interventions with a trade-off between beneficial and adverse effects	Interventions of unknown effect, including absence of reviews	Interventions likely to be ineffective or harmful

### Levels of delivery

The origin of evidence included the following three different levels of delivery of interventions and these were defined in the publication by the World Bank "Providing Interventions":

**(1) Community level/home**–health care providers at this level includes community health workers and outreach workers. It utilizes resources such as volunteers’ time, local knowledge, and community confidence and trust as channels for delivery of interventions generally related to safe motherhood, nutrition, and simple prevention and treatments. Many countries have attempted to construct links between community-based health care resources and households for a range of health programs. These programs do not substitute for a health system, but provide a channel for reaching families with information and resources. Community health workers (CHWs) not only promote healthy behaviors and preventive action but can mobilize demand for appropriate services at other levels. The success of community health efforts depends critically on the context, including level of development of infrastructure, services, and socioeconomic resources.

**(2) First level/outreach** - Health care providers at this level of care includes professionals, outreach workers as well as the community health workers. It includes a range of initiatives that are associated with the Alma Ata Declaration on Primary Health Care approved by WHO in 1978. More recently, the WHO Commission on Macroeconomics and Health described the need for developing services that are close to the client. The basic notion is a common one: recognition that a certain range of health care services must act as an interface between families and community programs on the one hand, and hospitals and national health policies on the other. There has been substantial convergence in the content of general first level primary care over time: maternity related care (for instance, prenatal care, skilled birth attendance, and family planning), interventions to address childhood diseases (such as vaccine preventable diseases, acute respiratory infections, diarrhea and prevention and treatment of major infectious diseases.

**(3) Referral level** - this level of delivery of interventions refers to hospitals in general. These can be either district hospitals or referral hospitals. The health care providers at this level are professionals.

***District hospitals* -** generally designed to serve people with services that are more sophisticated, technically demanding, and specialized than those available at a primary care facility/first level care, but not as specialized as those provided by referral hospitals. Their range of services includes diagnostics, treatment, care, counseling, and rehabilitation. District hospitals may also provide health information, training, and administrative and logistical support to primary and community health care programs. It concentrates skills and resources in one place for the delivery of interventions for conditions that are either uncommon or difficult to treat. It is also a repository of knowledge and diagnostic tools for assessing whether referral to an even more specialized facility is indicated.

***Referral hospitals***- referral hospitals provide complex clinical care interventions to patients referred from the community, primary/first, or district hospital levels. Referral hospitals need to provide many forms of support, including advice on which patients to refer, proper post discharge care, and long-term management of chronic conditions. Referral hospitals can also provide important managerial and administrative support to other facilities, serving as gateways for drugs and medical supplies, laboratory testing services, general procurement, data collection from health information systems, and epidemiological surveillance. They are also the vehicle for disseminating technologies by training new staff and providing continuing professional education for existing staff at different facilities.

## Data extraction and analysis

The review authors set up a triage process with standardized criteria for evaluating outputs from the search strategy and primary screening. Following an agreement on the search strategy, the abstracts (and the full sources where abstracts were not available) were screened by two abstractors to identify reviews adhering to the objectives. Any disagreements on selection of reviews between these two primary abstractors were resolved by the third reviewer. After retrieval of the full texts of all the reviews that met the inclusion/exclusion criteria, each review was double data abstracted into a standardized form. Information was extracted on the following criteria:

1. Characteristics of included reviews - description of each review included brief description of objectives, interventions, types of study design included, and outcomes reported;

2. Whether the review was a Cochrane or non-Cochrane review

3. And if they pooled the studies included.

Available systematic reviews were assessed for quality using the AMSTAR criteria (Assessment of the methodological quality of systematic reviews) [22]. Any disagreements were resolved by discussion and the final decision was taken by consensus within the team.

Over the next three papers we have discussed essential interventions for reproductive, maternal, neonatal and child health that can be delivered over the continuum of care. Table 3 has enlisted the interventions graded as A on the previously defined criteria at any level of health care delivery across the continuum of care.

**Table 3:** Grading of interventions according to the level of health care delivery

Intervention	Referral level	1^st^ level / outreach	Community
**Adolescents & pre-pregnancy**			

Family planning	A	A	A

Prevent& manage sexually transmitted illness including HIV for prevention and mother to child transmission for HIV and syphilis	A	A	A

Folic acid fortification and/or supplementation for preventing neural tube defects	A	A	A

**Pregnancy**			

Management of unintended pregnancy a) Availability and provision of safe abortion care when indicated and legally permitted b) Provision of post abortion care	A	B	-

**Appropriate antenatal care package:** Screening for maternal illness Screening for hypertensive disorders of pregnancy Screening for anemia Screening for fetal growth problems (IUGR) Iron and folic acid to prevent maternal anemia Tetanus immunization Counseling on family planning, birth and emergency preparedness Prevention and management of HIV, including with antireterovirals Prevent and manage malaria with insecticide treated nets and antimalarial Smoking cessation	A	A	C

Reduce malpresentation at term with external cephalic version	A	-	-

Management of pre-eclampsia • Calcium to prevent hypertension • Low dose aspirin to prevent hypertension	A	B	-

Magnesium sulphate for eclampsia	A	C	-

Induction of labor to manage premature rupture of membranes at term	A	-	-

Antibiotics for preterm rupture of membranes	A	B	-

Corticosteroids to prevent respiratory distress syndrome in newborns	A	-	-

**Childbirth**			

Prophylactic antibiotics for caesarean section	A	-	-

Management of postpartum hemorrhage (e.g. uterotonics, uterine massage)	A	B	C

Active management of third stage of labor to prevent postpartum hemorrhage	A	A	-

Cesarean section for absolute maternal indication	A	-	-

Induction of labor for prolonged pregnancy	A	-	-

Prophylactic uterotonics to prevent postpartum hemorrhage Management of postpartum hemorrhage (e.g. uterotonics, manual removal of placenta, uterine massage)	A	B	C

**Post natal (Mother)**			

Family planning	A	A	A

Prevent and treat maternal anemia	A	B	-

Detect and manage postpartum sepsis	A	B	-

Screen and initiate or continue antiretroviral therapy for HIV	A	A	-

**Post natal (newborn)**			

Immediate thermal care	A	B	B

Initiation of exclusive breastfeeding (within first hour)	A	A	A

Hygienic cord and skin care	A	B	B

Neonatal resuscitation with bag and mask (professional health worker)	A	B	-

Case management of neonatal sepsis, meningitis and pneumonia	A	B	-

Kangaroo mother care for preterm and for less than 2000g babies	A	B	-

Management of newborns with jaundice	A	B	-

Surfactant to prevent respiratory distress syndrome in preterm babies	A	-	-

Continuous positive airway pressure (CPAP) to manage babieswith respiratory distress syndrome	A	-	-

Extra support for feeding small and preterm babies	A	B	-

Presumptive antibiotic therapy for newborns at risk of bacterial infections	A	-	-

**Infancy and childhood**			

Exclusive breastfeeding for 6 months	A	A	A

Continued breastfeeding and complementary feeding from 6 months	A	A	A

Prevention and case management of childhood malaria	A	A	A

Vitamin A supplementation from 6 months of age	A	A	A

Comprehensive care of children infected with or exposed to HIV infection	A	A	-

Routine Immunization and H. influenzae, meningococcal, pneumococcal and Rota virus vaccines	A	A	B

Management of severe acute malnutrition	A	A	-

Case management of childhood pneumonia	A	A	A

Case management of diarrhea	A	A	A

**Cross cutting community strategies**			

Home visits for women and children across the continuum of care	-	-	A

## Conclusion

Poor maternal, newborn and child health remains a significant problem and represent two of the most difficult to achieve targets among the MDGs particularly in LMICs. Majority of maternal deaths occur during pregnancy and childbirth, while the risk of infant’s death is highest in the first 28 days of life. The situation is grave in Asia and Sub-Saharan Africa, where mortality among mothers and neonates is the highest in the world. The rate of maternal mortality is 129 times and rate of under-five mortality is 71 times higher in LMIC compared to high income countries. Several factors contribute to poor maternal, newborn and child deaths; and with simple, low cost interventions, these deaths can be avoided particularly in low income countries. Furthermore, the health and well-being of mothers and infants are closely linked; the interventions for improving women health have beneficial impacts on birth and neonatal outcomes. The aim of this exercise is to develop consensus on the content of RMNCH packages of interventions at each level of the health system across the continuum of care. With this rationale, a total of 142 RMNCH interventions were identified from several recent relevant bodies of work. Of these, 56 essential interventions were short listed based on the evidence of their efficacy, effectiveness and impact on survival; their suitability for implementation in low- and middle- resource settings and their likelihood to be delivered through the health sector from the community to the referral. These were further classified and allotted to different plausible level of health system delivery levels.

This introductory paper helps understand the background and methodology in depth for the work which has been undertaken and detailed over next few papers. This series, in whole, is highlighting the essential reproductive, maternal, newborn and child health interventions and their effectiveness for maternal, fetal, neonatal and child health. The last paper of this series is summarizing the delivery of these essential interventions as per the level of health care and in the form of care packages.

## Competing interests

We do not have any financial or non-financial competing interests for this review.

## Peer review

The reviewer reports for this article can be found in Additional File 1.

## Supplementary Material

Additional file 1 Peer review. Click here for file

## Declarations

This article has been published as part of *Reproductive Health* Volume 11 Supplement 1, 2014: Essential intervention for maternal, newborn and child health. The full contents of the supplement are available online at http://www.reproductive-health-journal.com/supplements/11/S1. Publication charges for this collection were funded by the Partnership for Maternal, Newborn & Child Health (PMNCH).
