# Supplementary material for: Essential interventions for maternal, newborn and child health: background and methodology
Source: Reprod Health. 2014 Aug 21;11(Suppl 1):S1. doi: 10.1186/1742-4755-11-S1-S1 (PMC4145855; doi:10.1186/1742-4755-11-S1-S1)
Supplement: Additional file 1 — Peer review. [file 1742-4755-11-S1-S1-S1.pdf]

Referee's comments to the authors– this sheet WILL be seen by the author(s) and published with the article

Title	**Essential interventions for maternal, newborn and child health: Background and Methodology**
Author(s)	Zohra S Lassi, Rehana A Salam, Jai K Das, Zulfiqar A Bhutta
Referee's name	Ana Pilar Betran Lazaga

When assessing the work, please consider the following points, where applicable:

1. **Is the question posed by the authors new and well defined?**
2. **Are the methods appropriate and well described, and are sufficient details provided to replicate the work?**
3. **Are the data sound and well controlled?**
4. **Does the manuscript adhere to the relevant standards for reporting and data deposition?**
5. **Are the discussion and conclusions well balanced and adequately supported by the data?**
6. **Do the title and abstract accurately convey what has been found?**
7. **Is the writing acceptable?**

Please make your report as constructive and detailed as possible in your comments so that authors have the opportunity to overcome any serious deficiencies that you find and please also divide your comments into the following categories:

- Major Compulsory Revisions (which the author must respond to before a decision on publication can be reached)
- Minor Essential Revisions (such as missing labels on figures, or the wrong use of a term, which the author can be trusted to correct)
- Discretionary Revisions (which are recommendations for improvement but which the author can choose to ignore)

Where possible please supply references to substantiate your comments.

When referring to the manuscript please provide specific page and paragraph citations where appropriate.

General comments: This is an introductory manuscript describing the objectives and methodology used in other three papers related to this. I understand the four papers will be published together so this first manuscript will be read with the information of the others at hand which will make it more clear. The manuscript is well written and straight forward, particularly the introduction. Minor comments for the authors are presented below.

Major compulsory revisions:

Minor essential revisions:

1. **Similar exercises of compilation of effective strategies and interventions for maternal and child survival and means of distribution have been previously published (e.g. Lancet Maternal Survival Series, 2006; Adam et al. Cost effectiveness analysis of strategies for maternal and neonatal health in developing countries. BMJ 2005;331:1107). Is the purpose of this manuscript presenting an update or it includes other type of analysis, perspective or other issues contemplated? It would be useful for the readers to understand this point.**
2. **The abstract has a typo: "… This paper is be an introductory…"**

Continued:

3. **Page 2, paragraph 1: Could author reference the last sentence? Is it reference number 2?**
4. **Page 2, paragraph 2: The maternal mortality ratios referenced are from a publication in 2006. Latest maternal mortality estimates from WHO/UNICEF/UNFPA were published in 2012 by the WHO (http://whqlibdoc.who.int/publications/2012/9789241503631_eng.pdf). In this publication, the Maternal mortality ratio (MMR) of sub-saharan Africa is 500 per 100,000 live births (not 50 as stated in the manuscript). It is probably a typo but the other MMRs could be revised according to the WHO more updated estimates.**
5. **Page 4: Selection of Interventions. The paragraph starts by saying "The interventions were prioritized..." In this manuscript, this is the only reference to the priotirization. It would be more clear to the reader if authors explained why the prioritization statement is included here or with what objective. Are the other three papers going to present a list of interventions in an recommended order? Also, the paragraph with the heading "Selection of the interventions" talks about the prioritization whereas the selection seems to be the previous paragraph where the inclusion criteria is detailed.**

Referee's comments to the authors– this sheet WILL be seen by the author(s) and published with the article

Title	**Essential interventions for maternal, newborn and child health: Background and Methodology**
Author(s)	Zohra S Lassi, Rehana A Salam, Jai K Das, Zulfiqar A Bhutta
Referee's name	Andre Lalonde

When assessing the work, please consider the following points, where applicable:

1. Is the question posed by the authors new and well defined?
2. Are the methods appropriate and well described, and are sufficient details provided to replicate the work?
3. Are the data sound and well controlled?
4. Does the manuscript adhere to the relevant standards for reporting and data deposition?
5. Are the discussion and conclusions well balanced and adequately supported by the data?
6. Do the title and abstract accurately convey what has been found?
7. Is the writing acceptable?

Please make your report as constructive and detailed as possible in your comments so that authors have the opportunity to overcome any serious deficiencies that you find and please also divide your comments into the following categories:

- Major Compulsory Revisions (which the author must respond to before a decision on publication can be reached)
- Minor Essential Revisions (such as missing labels on figures, or the wrong use of a term, which the author can be trusted to correct)
- Discretionary Revisions (which are recommendations for improvement but which the author can choose to ignore)

Where possible please supply references to substantiate your comments.

When referring to the manuscript please provide specific page and paragraph citations where appropriate.

General comments:The article is well written and makes a good resume of major issues in MNCH.

Major compulsory revisions:

Minor essential revisions:Page 2 :Add "care" after the words maternal health.
MM in sub Sahara Africa is not 50 but 500 per 100,000. Also when quoting MM ratio and ot total number of deaths always include +or – 20%..
Page 3end of para top pf page..add "comprehensive" after the words basic obs care.
Page 3 1st para..example of fetoscope to reduce neonatal deaths is not appropriate..need another example..
Box 2 .Under Bith..under community it includes prophylactic antibiotics for C section..C-Section would not occuer at this level.. PPH management under community should be a "B: as treatment with Misoprostol has been shown to be possible and effective at community level at the time and during

(continue on the next sheet)

Continued: preparation and referral. AMSTL under community needs to be a “B” as research has shown this to be positive..especially since the oxytocic is the main component of AMSTL..Again Misoprostol has been shown to be effective at community level.in use of AMSTL
